# Family Resilience, Support, and Functionality in Breast Cancer Patients: A Longitudinal Pre- and Post-Operative Study

**DOI:** 10.3390/bs15070880

**Published:** 2025-06-27

**Authors:** Dimitrios Charos, Maria Andriopoulou, Giannoula Kyrkou, Maria Kolliopoulou, Anna Deltsidou, Anastasia Bothou, Victoria Vivilaki

**Affiliations:** 1 General Anti-Cancer Oncology Hospital Agios Savvas, 11522 Athens, Greece; 2 “Konstantopouleio” General Hospital of Nea Ionia, 14233 Athens, Greece; 3 Midwifery Department, University of West Attica, 12243 Athens, Greece

**Keywords:** breast cancer, family functionality, family dysfunction, family support, family resilience, pre operative, post operative

## Abstract

This longitudinal study investigated changes in family resilience, support, and functionality among breast cancer patients during the pre-operative and post-operative phases. The study was grounded in McCubbin’s model, emphasizing the psychosocial impact of illness and the cultural dynamics of the Greek family system. A longitudinal cohort study was conducted on women diagnosed with breast cancer, aged over 18, undergoing mastectomy, fluent in Greek, and capable of completing questionnaires at two time points, pre- and post-operatively. Standardized instruments were used: the Family Assessment Device (FAD), the Family Crisis Oriented Personal Evaluation Scales (F-COPES), the Family Problem Solving Communication Scale (FPSC), and the Family Support Scale (FS-13). Data were analyzed using paired *t*-tests, ANOVA, and partial correlations. The sample consisted of 58 women with breast cancer. The mean age of participants was 52 years. According to post-operative measurements, the scales that had a significant change were FS-13 (change −12 and SD = 6.9, paired *t*-test, *p* < 0.001), and the subscale “Problem Solving” of FAD (change 0.13 and SD = 0.44, paired *t*-test, *p* = 0.048). The remaining scales did not change significantly post-operatively. Depending on the type of treatment, the scales that had a significant difference post-operatively regarded the participants, who had undergone chemotherapy in the dimension “Acquiring Social Support” (ANOVA = 0.173, *p* = 0.030), the participants, who had undergone radiotherapy in the dimension “Seeking spiritual support” (ANOVA = 0.122, *p* = 0.035), and in the FS-13 scale across all treatment types (ANOVA = 0.458, *p* < 0.001). The post-operative phase of breast cancer was a psychosocially vulnerable time, accompanied by an increase in family dysfunction and a significant decrease in family support across all types of treatments, while patients receiving chemotherapy or radiotherapy experienced a corresponding decrease in social and spiritual support. Finally, enhanced family support was associated with improved family resilience, highlighting the need for targeted psychosocial interventions during this period.

## 1. Background

The incidence of breast cancer has risen dramatically in recent years. In 2020, 2.3 million new cases were recorded, and this number is projected to increase to 3.2 million by 2050. It is currently the leading cause of cancer-related death among women in 40 European countries (Sung et al., 2021).

The role of family, significant others, and social support is vital in accepting and adapting to illness, supporting and empowering patients, and fostering family resilience (Andriopoulou et al., 2018).

Recent research has highlighted that breast cancer affects both patients and their close relatives from the moment of diagnosis. Breast cancer disrupts family dynamics, including role distribution, as well as relationships with their partner/spouse, children, extended family, social networks, and social support (Segrin et al., 2018; Altun et al., 2019; Charos et al., 2023).

Some families demonstrate resilience that enables them to recover from the crisis brought on by the disease. This phenomenon has inspired researchers to explore how resilience within the family contributes to individual well-being. According to recent studies, family resilience enhances individual resilience and reduces caregiver burden (Y. Li et al., 2018; Y. Liu et al., 2018; K. W. S. Li et al., 2019; Faccio et al., 2019; Xu et al., 2021); fosters the strengths of the family structure, such as communication, stability, and roles (Coyne et al., 2012); and promotes patients’ ability to maintain their interpersonal relationships and manage diagnosis and treatment more effectively (Y. Liu et al., 2018). The concept of family resilience is especially significant, as it reframes the family not as inherently dysfunctional but as a system capable of adapting to adversity by drawing on its internal strengths and fostering mutual growth among its members (Walsh, 2004; Faccio et al., 2019).

One of the key theoretical frameworks for family resilience is the FAAR (Family Adjustment and Adaptation Response) model developed by McCubbin and Patterson (1983), based on Hill’s theory of family stress. This model was developed to understand how families cope with crisis situations and emphasizes the interplay of factors such as family functioning, coping mechanisms, and resilience in coping with stress and in the family’s adjustment after the stressful event.

According to this model, when a family faces a crisis (such as a diagnosis of cancer), two phases are activated: the Adjustment Phase, in which the family attempts to manage the situation with existing resources and strategies, and the Adaptation Phase, in which the crisis is prolonged and the family is forced to readjust roles, communication patterns, and problem-solving strategies.

Successful adaptation relies on the mobilization of internal and external resources, such as family cohesion and social support.

In the context of breast cancer, especially during the pre- and post-surgical period, changes in the cohesion and roles of family members are observed, and McCubbin’s model is valuable for understanding the reactions of family structure and adaptation after the disease.

Concurrently, social support is recognized as a crucial factor in managing illness, facilitating coping processes, and promoting successful adaptation (Merluzzi et al., 2016; Charos et al., 2021), improving patients’ mental health as well as their quality of life (Schleife et al., 2012).

Although the needs of the entire family system are particularly increased after a breast cancer diagnosis, these needs are often neglected, and psychosocial support services need restructuring (Hammersen et al., 2021; Moshtagh & Allahyari, 2022).

Targeted interventions aimed at improving intra-family communication and functioning contribute to improving family relationships and increasing patients’ quality of life (John et al., 2013; Altun et al., 2019; Hammersen et al., 2021; Charos et al., 2023).

The pre-operative and post-operative periods are critical for both patients and their families due to the high vulnerability of patients, which has not been extensively studied. During this period, patients—mainly due to the mastectomy and the beginning of treatment—experience high rates of worry, anxiety, difficulty adjusting, fear of treatment, fear of recurrence, etc. (Nakatani et al., 2013; Kovac et al., 2014). A decline in support, an increase in post-surgery dysfunction, and a greater need for psychosocial support are expected. Temporal changes are expected to directly affect perceived support, coping strategies, and family functioning.

Despite the abundance of studies that highlight the importance of family support in cancer patients, most existing studies are either cross-sectional or focus exclusively on the psychological state of patients, neglecting the family as a systemic unit of support and resilience, or study patient survivors (B. Liu et al., 2021; Xu et al., 2021; Chen et al., 2021; Acquati et al., 2020; Naidoo & Greeff, 2022). Longitudinal studies that focus on the course of family dynamics and family resilience during the pre-operative and post-operative periods remain extremely limited. Furthermore, the impact of treatment type on patients’ perceptions of family support and functionality has been poorly researched.

The cultural dimension, and especially the case of the Greek family, which concerns a context where the family takes an active role in supporting patients, has not been sufficiently studied (Kalogeraki, 2009; Andriopoulou et al., 2018). The majority of studies concern Northern European/American data, while Southern Mediterranean societies such as Greece, which use different definitions of “resilience” (Segrin & Badger, 2010), have not been extensively studied (Kalogeraki, 2009; Segrin & Badger, 2010).

Our study is based on the FAAR model of McCubbin and Patterson (1983), and uses four research tools that cover different but complementary aspects of family adjustment, and captures changes in family support and functionality in breast cancer patients in Greek society.

This study investigated changes in family functionality, support, and resilience in patients with breast cancer during the pre- and post-surgical period. It is based on McCubbin’s FAAR model, while also considering the cultural characteristics of the Greek family system.

## 2. Materials and Methods

### 2.1. Study Design and Sample Collection

This study investigated changes in key dimensions of the McCubbin model that define family resilience: family functioning (FAD), family support (FS-13), family problem-solving and communication (FPSC), and family coping strategies in crisis situations (F-COPES).

According to the model, the family mobilizes internal and external protective resources and adaptation strategies in order to maintain or regain its functionality after a stressful event, and is influenced by the cultural context of each society.

More specifically, the variables of this study were:

1. Stressful event: the diagnosis of breast cancer in combination with surgery.
2. Family resources: communication, support, and roles.
3. Family perceptions: the interpretation of stress and meaning-making strategies.
4. Resilience/adaptation: the outcome of the process.

The study’s assessment at two time points reflects the transition from the immediate adaptation phase to the resilience testing phase. The study takes into account the connection between family culture and psychosocial resilience.

**Figure 1:**
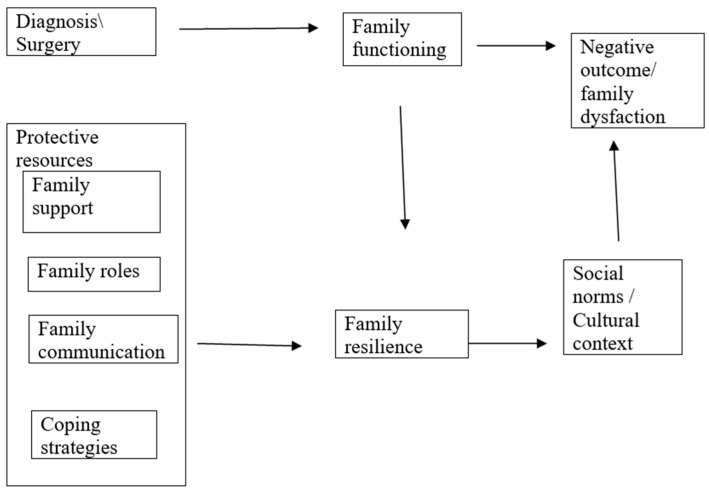
Linking study variables and the theoretical model (adapted and developed by the authors based on McCubbin model).

The diagram below describes the connection between the study variables and the theoretical framework (Figure 1).

The study was a longitudinal study focused on the initial phase of breast cancer disease. The study was conducted in two time periods in the pre-operative and post-operative stages of the disease through the administration of validated scales. The first administration was performed in the surgical clinic when they were admitted for surgery. The second administration was carried out at about three months, a time period when the women had already started their treatments. In this period of time, patients are at the beginning of psychological management and awareness of their illness and treatment.

The administration of the questionnaires at the pre-operative stage took place in person. A full briefing on the purpose and type of study was conducted, and written informed consent was then obtained from the patients for their participation in the study. At the same time, a supplementary guide form was provided to facilitate the completion of the questionnaire, and the submission of complaints and comments was encouraged. It was also made clear that participants retained the right not to participate in the study and to discontinue their participation without any impact on the care provided in their treatment.

After about two months, the participants were contacted in order to determine the second completion of the questionnaire. All participants who completed the study questionnaire within three months were included in the sample. Participants who were too late to respond (after three months) were excluded. The waiting period for the top-up was a week from the digital update. The questionnaire was administered in digital format.

This study was reported in accordance with the STROBE (Strengthening the Reporting of Observational Studies in Epidemiology) guidelines.

### 2.2. Measures

The research instrument consists of two sections. The first section included the demographic and social characteristics of the sample, and the second section included scales validated for the Greek population.

#### 2.2.1. Sociodemographic Characteristics

This section included self-reported variables such as age, marital status, education level, employment status, place of residence, monthly income, motherhood, and family history of cancer. The purpose was to capture the participants’ sociodemographic and clinical profiles.

#### 2.2.2. Family Assessment Device (FAD)

The FAD created by Epstein et al. (1983), according to the McMaster model, is a multidimensional scale that covers the whole range of family functioning. It consists of 60 questions in the form of a 4-Likert scale and four gradations from strongly agree to strongly disagree (1 = strongly agree to 4 = strongly disagree). It includes six subscales overlapping the six orders of the McMaster model, such as: (a) problem solving, (b) roles, (c) communication, (d) emotional responsiveness, (e) emotional involvement, (f) behavioral control, and (g) general functioning. Example item: “In times of crisis, we can turn to each other for support.” Higher scores indicate greater family dysfunction. The subscale score is the mean of all items. Cronbach’s α varies between 0.72 and 0.92. In the Greek language, it was validated by Tsamparli et al. (2018).

#### 2.2.3. Family Crisis Oriented Personal Evaluation Scales (F-COPES)

The F-COPES was created by McCubbin et al. (2001) and is based on the family resilience and adaptation model. It measures crisis in the family and identifies the strategies the family demonstrates to manage a problem. It consists of 30 items rated on a 5-point Likert scale (1 = Never to 5 = Always), covering five subscales: acquiring social support, reframing, seeking spiritual support, mobilizing the family to accept help, and passive appraisal. Example item: “Seek spiritual support.” Subscale and total scores are calculated by summing responses. Higher scores indicate more frequent use of coping strategies. In Greek, it was validated by Gouva et al. (2016), and Cronbach’s αwas 0.77.

#### 2.2.4. Family Problem Solving Communication (FPSC)

The FPSC was created by McCubbin et al. (1996a, 1996b, 2001) and assesses the ability of family communication to resolve family problems and conflicts with the ultimate goal of measuring family stress and family resilience. It comprises 10 items, rated on a 5-point Likert scale (1 = strongly disagree to 5 = strongly agree). Example item: “Family members listen to each other’s opinions.” The mean score reflects the quality of communication and problem-solving in the family. The FPSC had a Cronbach’s α of 0.89.

#### 2.2.5. Family Support Scale (FS13)

The FS13 was created by Julkunen and Greenglass (1989) and validated in the Greek population by Tselebis et al. (2011). It is a self-report scale that aims to assess the patient’s perception or subjective sense of family support. It includes 13 items, each rated on a 5-point Likert scale (1 = strongly disagree to 5 = strongly agree). Example item: “I feel that my family helps me during my illness.” The total score ranges from 0 to 39. Higher scores represent stronger perceived family support. The Greek validation reported a Cronbach’s α of 0.82 (Tselebis et al., 2011).

### 2.3. Participants

This study was conducted in three surgical clinics of public hospitals in Athens, from January 2020 to December 2021. From a random sample of 160 women with a recent diagnosis of breast cancer, who consented to take part in the research, only 72 met the criteria, and 58 women finally answered. The response rate was a satisfactory 80%, considering the adverse conditions created by the COVID-19 pandemic. All participants completed both the first and second phases of the study.

The inclusion criteria were:

- Patients with a recent breast cancer diagnosis
- Age over 18 years old
- Be able to respond to the research tool by digital means
- Patients were admitted to hospital and underwent mastectomy
- Informed consent for participation
- Being fluent in Greek
- Being literate

Participants who did not strictly meet the selection criteria were excluded. More specifically, the exclusion criteria were participants with metastatic cancer, psychiatric comorbidity, or inability to complete Greek-language questionnaires.

For the cultural adaptation of the questionnaire, the scales were administered to a small sample of patients, and a pilot study was conducted on a small sample of patients.

Participants were followed up three months after surgery via online questionnaires, ensuring consistent follow-up procedures.

Although no formal power analysis was performed, the sample size was considered sufficient to detect moderate to large within-group differences between the two time points. This assessment was based on the longitudinal nature of the study and the limitations imposed by the COVID-19 pandemic, which made it difficult to recruit and follow up participants.

### 2.4. Ethics

Participants completed their questions anonymously, and sensitive personal data were secured. The rules and guidelines set by the EU General Data Protection Regulation (2016), along with the relevant Greek legislation and the decisions of the Personal Data Protection Authority, were followed, as well as the Declaration of Helsinki (2013). Thus, the risk of data leakage, which could damage the personal, social, cultural, and economic status of the subjects, was prevented.

Efforts to reduce potential bias included the use of validated scales, standardized administration procedures, and exclusion of patients who responded late after three months post-operatively.

## 3. Statistical Analysis

Using the Kolmogorov–Smirnov test, the distributions of the quantitative variables were tested for normality. For variables that were normally distributed, mean values and standard deviations (standard deviation = SD) were used for their description, while for those that were not normally distributed, medians and interquartile ranges were additionally used. Absolute (N) and relative (%) frequencies were used to describe qualitative variables.

To test the relationship between two quantitative variables, Pearson’s correlation coefficient (r) was used. A paired *t*-test was used to compare scales between measurements. Repeated-measures analysis of variance (ANOVA) was used to test for differences in pre- and post-surgery scales by type of treatment.

Also, with the above method, it was assessed whether the degree of change over time of the parameters under study was different between the types of treatment. The degree of correlation between changes in the scales was tested with partial correlations, taking into account the treatment the participants received.

The internal reliability of the questionnaire was tested using the Cronbach α coefficient. The significance level was set at 0.05. The statistical program SPSS 22.0 was used for the analysis.

## 4. Results

### 4.1. Descriptive Statistics of the Sample

In this study, the sample consisted of 58 patients with breast cancer. The mean age of the participants was 52.1 (SD = 10.3 years) (Table 1).

According to Table 1, the majority of patients’ educational level (32.8%) was higher education. The largest percentage of the participants (29.8%) were public employees, followed by 21.1% private employees.

Regarding marital status, 66.7% of patients were married, and the vast majority of participants were mothers (82.8%). The mothers in the sample who had protected members corresponded to 34.4%.

Regarding family history of cancer, 55.2% of the patients had a history of cancer in their family, and the largest percentage had breast cancer (37.9%).

Patients first informed their spouse/partner about their diagnosis (56.9%), followed by their children (12.1%). People who were close to the patients during their illness were mainly their spouse/partner (51.7%), followed by their children (24.1%).

### 4.2. Measurements and Changes in Scales After Surgery

The following table shows the scores on the studied scales before and after the operation (Table 2).

The score on the “Problem Solving” dimension increased significantly after surgery, indicating greater family dysfunction. In contrast, the score on the FS-13 family support scale decreased significantly, indicating a significant decrease in the family support participants received after surgery.

The remaining scales did not change significantly post-operatively.

### 4.3. Changes in Scales According to the Type of Treatment

Participants’ scores on the FAD scale did not differ significantly by type of treatment, either before or after surgery. Also, the scores on the dimensions of this scale did not change significantly post-operatively in any type of treatment.

The changes in the F-COPES scale, depending on the type of treatment of the participants, are given in the following table (Table 3).

After the Bonferroni correction, no significant difference was found in the score of the “Reframing” dimension between treatment types. Similarly, after surgery, no significant differences were found between types of treatment in scores on the dimensions “Mobilizing Family to Acquire and Accept Help” and Overall F-COPES.

There was a significant decrease in the score of chemotherapy patients on the “Acquiring Social Support” dimension, suggesting that chemotherapy patients receive less social support from relatives, friends, and extended family.

Similarly, there was a significant decrease in the score of women who had undergone radiation therapy on the “Seeking Spiritual Support” dimension, indicating reduced seeking of spiritual support.

The degree of change in F-COPES scores was similar across participants’ treatment types.

The changes in the FS-13, FPSC scales according to the type of treatment of the participants are given in the following table (Table 4).

From the table above, it can be seen that the scores of the participants did not differ significantly between the types of treatment, neither before nor after the operation.

Of particular note, after surgery, there was a significant reduction in the FS-13 scale across all treatment types. This suggests that patients felt they received much less family support during their treatment.

The remaining scales remained unchanged. The degree of change in scores was similar across treatment types.

### 4.4. Correlations Between Scale Changes

Considering participants’ type of treatment, no significant correlations (partial correlation coefficients) were found between changes in FAD and F-COPES scales.

Regarding the partial correlation coefficients of the changes in the FS-13 and FPSC scales with the change in the FAD scale, it is found that the change in the “Behavioral Control” dimension was significantly negatively related to the change in the “Family Support scale” (FS-13) (r = −0.60, *p* = 0.038). Therefore, improving family support among participants reduced family dysfunction.

The following table describes the partial correlation coefficients of the changes in the FS-13 and FPSC scales with the change in the F-COPES scale, taking into account the type of treatment of the participants (Table 5).

Change in the “Reframing” dimension was significantly positively related to change in the “Family Support” scale. Change in the F-COPES total score was significantly positively related to change in the “Family Support” scale.

Therefore, the more the patients experienced increased family support, the more they sought social support, reframed stress, and, in general, improved the management of the crisis in the family. Consequently, improving family support increased family resilience.

## 5. Discussion

This longitudinal study included 58 breast cancer patients who underwent mastectomy. Their mean age was 52.1 (SD = 10.3 years).

Post-operative results indicated significant changes in family support and the FAD Problem Solving subscale. The remaining scales (F-COPES, FPSC) did not change significantly post-operatively. Based on the findings of the study, family resilience was not significantly affected from the first measurement (pre-operative period) to the second measurement (post-operative period), in contrast to family support and family dysfunction, which were affected.

Post-operative family support declined significantly, though multiple studies highlight its benefits for patients, such as adaptability, better management of stress and illness, improved quality of life, and mental and family resilience (Yoo et al., 2014; Y. Liu et al., 2018; B. Liu et al., 2021; Xu et al., 2021).

The score on the “Problem Solving” dimension increased significantly, indicating greater family dysfunction following the illness. Recent research suggests that breast cancer often exacerbates existing dysfunctional and conflictual family relationships (Segrin et al., 2018).

Depending on the type of treatment, it was observed that family functionality was not affected by the treatments the patients underwent. Similar studies in breast cancer patients have shown that family functionality is not observed to be significantly associated with the disease and treatment. Problems within families are manifested by the existing dysfunctional relationships between members (Stefanou et al., 2020; Tavares et al., 2018; Altun et al., 2019). Breast cancer patients who were mothers seemed to have tried to readjust their parental role, strengthening constructive communication, the cohesion of family relationships, etc. (Xu et al., 2021). A small proportion of children have increased levels of stress and psychosocial problems following their mother’s illness (Bultmann et al., 2014). Effective, mutual, and constructive communication increases the quality of life, and the family invents effective coping strategies for the situation that the disease creates in the family system (Acquati et al., 2020).

Depending on the type of treatment, there was a significant decrease in the score for the strategy “Acquiring Social Support” among patients who had undergone chemotherapy. This suggests that patients who have undergone chemotherapy receive less social support from relatives, friends, and their extended family. A recent study concluded that breast cancer patients who had less social support had more side effects from chemotherapy (Oh et al., 2020). A similar study highlights that chemotherapy causes anxiety in patients, while social and family support reduces the distress caused by chemotherapy (Charalambous et al., 2017).

Similarly, patients who underwent radiation therapy showed a reduced tendency to seek spiritual support. Similar studies conclude that spirituality supports patients and increases their resilience (Naidoo & Greeff, 2022; Fradelos et al., 2018). Therefore, the treatment of breast cancer patients partially affects family crisis management strategies, which affects their family’s resilience and adaptability. Chemotherapy and radiation affect the family resilience of patients. Two cross-sectional studies conclude that strengthening family resilience empowers individual resilience, quality of life, and burden in both caregivers and breast cancer patients (Y. Li et al., 2018; Y. Liu et al., 2018).

Family support decreased significantly across all types of treatments, suggesting that patients felt they received much less family support during their treatment than at the beginning of their illness diagnosis. In addition, the type of treatment patients received did not affect their ability to solve family problems.

Therefore, considering treatment type, family resilience and family support were negatively affected. In contrast, family dysfunction was not affected.

Consequently, chemotherapy consistently reduces family support, radiotherapy appears to influence spiritual support-seeking behaviors, and hormone therapy exhibits slightly different trends, possibly due to its less invasive nature.

These findings suggest that the type of treatment likely plays a different role in shaping family coping strategies and support needs, justifying individualized and targeted psychosocial interventions.

Taking into account the type of treatment the participants received, the significant correlations between scale changes in the post-operative period indicated that the more patients had increased family support, the more they used the strategy of seeking social support, reframing, and stress management, and overall better managed the crisis in the family that cancer triggered.

Consequently, improving family support for breast cancer patients increases family resilience. The findings of this study are consistent with the findings of the study by Chen et al. (2021).

### 5.1. Cultural Context of the Greek Family

It is important to take into account the cultural context in which the findings of this study are interpreted and to integrate them into the cultural context of Greek society. The Greek family is characterized by strong emotional bonds and is the main source of support, especially in conditions of illness (Kalogeraki, 2009). The family in Greece fully assumes the support of patients with cancer (Andriopoulou et al., 2018). The diagnosis of breast cancer does not only concern the patient, but the entire family network.

The significant decrease in family support observed post-operatively can be interpreted as a reflection of fatigue, emotional distress, or difficulty adapting to new family balances and role changes, and not as rejection or indifference. Also, the strong connection between family support and adaptation strategies demonstrates that family culture decisively influences the patient’s psychosocial resilience.

### 5.2. Original Contribution and Research Gap

This study has addressed a significant gap in the literature regarding how family functioning, family support, and resilience change in breast cancer patients during the transition from pre-operative to post-operative periods. While most studies are cross-sectional or focused exclusively on the patient, this work was based on a longitudinal approach, utilizing the FAAR model of McCubbin and Patterson (1983) while also taking into account the cultural specificity of the Greek family.

As noted above, the results confirmed that family support decreases significantly after surgery. This finding, which is in agreement with studies such as those by Y. Liu et al. (2018) and Xu et al. (2021), is particularly important, as it is associated with the strengthening or weakening of adaptation strategies (e.g., seeking social support, reinterpretation). In terms of family functioning, a deterioration in problem-solving was recorded, which reinforces the view that surgery burdens dysfunctional family dynamics (Segrin et al., 2018).

Of particular interest are the findings in relation to the type of treatment. Chemotherapy was associated with a decrease in perceived social support, while radiotherapy was associated with a decrease in spiritual support. These differences highlight the need for personalized psychosocial support interventions, depending on the type of treatment, as already highlighted in the study by Charalambous et al. (2017). On the contrary, family functioning did not appear to be affected by the type of treatment, reinforcing the theory that dysfunction has a pre-existing basis and is not caused exclusively by the disease (Tavares et al., 2018).

The study also highlighted the importance of the cultural dimension. In the Greek context, the family is traditionally the main source of support. The post-operative decline in support is likely not due to rejection, but to emotional exhaustion and difficulty in re-defining roles. These results confirmed that family support is inextricably linked to family resilience and crisis management capacity.

In conclusion, the study has reinforced the need to design specialized, culturally sensitive psychosocial interventions, especially during the vulnerable post-operative period. At the same time, the results emphasized the need to re-examine the role of the family as a key variable in oncology care services, not only as a means of support but also as an active subject of intervention. The diachronic, culturally based design offers us a new lens through which we can understand family resilience in the context of breast cancer.

The contribution of the study lies in its longitudinal design, the integration of four family dynamics assessment tools, and the connection of the findings to psychosocial care in the Greek cultural context—thus offering a new perspective on understanding family resilience to breast cancer.

### 5.3. Clinical Implications

The ultimate goal of the study was the clinical impact of the results. The results of this study highlighted the need for targeted clinical interventions, especially during the post-operative period. Given the significant decrease in family support, the impact of family functionality and resilience during the post-operative period,

- The establishment of specialized psycho-oncology centers in public hospitals, which would provide systematic monitoring of psychosocial needs and would provide targeted support during critical phases of treatment for patients and their families.
- Utilization of digital health infrastructure for psychosocial interventions through digital media, especially in remote areas.
- Empowerment of both patients and their families. Strengthening family resilience, social support, and family support in patients at increased risk.
- Training health professionals in the recognition and management of the psychosocial dimension of cancer, based on the findings of the study.

Although our study focused on women with breast cancer, we recognize the need for future research in male breast cancer patients. Although rare, breast cancer in men has equal psychological and social impacts on both themselves and their families. Men may have more difficulty expressing their need for support due to societal stereotypes.

Future studies would be advisable to design longitudinal studies, not only for the patients, but also for other members of their families, such as spouse/partner, children, for a longer period of time (six months, one year, etc.), and in a larger sample.

The design of mixed-method qualitative and quantitative studies, studies with targeted psychosocial interventions, and finally, the inclusion of other variables, such as quality of life, caregiver burden, cross-cultural factors, etc., would also be suggested.

### 5.4. Generalizability of the Findings

An important issue that must be taken into account is the generalizability of the study results. First, this study was conducted in three large hospitals in Attica, where patients from various social and geographical groups (urban, semi-urban, and island environments) are addressed, thus enhancing the representativeness of the sample for the Greek reality.

Second, the longitudinal design, the use of four valid tools, and the assessment at two critical time points (before and after surgery) enhance the transferability of the findings to other cancer patient populations.

Third, the culture of the Greek family, which is based on strong ties and intense care, is a critical factor that differentiates the results in relation to countries with more individualistic standards.

Nevertheless, the study has offered useful comparisons for other Mediterranean or traditional societies with similar family structures.

Finally, although generalizability to other populations outside of Greece must be approached with caution due to cultural differences, the theoretical focus (FAAR model) offers a flexible framework that can be applied to other cultural environments.

### 5.5. Limitations and Strengths of the Study

This study was conducted during the COVID-19 pandemic, resulting in increased study limitations. Initially, it contributed to the difficulty of collecting the sample and at the same time prevented any information from other family members.

The second limitation concerns the small sample size. However, under the circumstances of the COVID-19 pandemic, and the specificity of the study (long-term and with follow-up), it provides sufficient power to detect mean differences, especially when the effect sizes are large (as in FS-13), and the study can be repeated in the future.

A third limitation concerns the time stages of the study (pre-operative and post-operative). The limitations of the study can be suggestions for new studies in the future.

However, despite the limitations, the study is also notable for its strengths. This study highlighted important characteristics, mainly in terms of the combination of variables and the time period over which the study was conducted. The pre-operative and post-operative periods of breast cancer patients are vulnerable times that have not been extensively studied by the scientific community.

The sample of participants was taken from three major breast cancer centers in the capital, where all social strata and regions of the country have access, and the response rate of the study was very satisfactory.

This study was based on the FAAR model, utilizing four research tools that covered different but complementary aspects of family adjustment: FAD, FS-13, F-COPES, and FPSC. It is one of the few longitudinal studies to capture changes in family support and functioning in breast cancer patients in Greek society.

It focused on how the disease and treatment affect family dynamics at two critical time points (pre- and post-operative). It has provided empirical data that can be used to develop culturally sensitive psychosocial support interventions.

Therefore, this particular study contributes significant benefits to the scientific community, both for patients suffering from breast cancer and for their family and social network, in a vulnerable and critical period of their disease, such as the pre-operative and post-operative period. At the same time, the study expects to give impetus to the scientific community for further study, with the ultimate goal of improving the quality of psycho-oncology services.

The use of digital media is an important innovation in patient support, especially in Greece, which is an island country with many remote places.

## 6. Conclusions

Based on the findings of the study, post-operative breast cancer patients experienced greater family dysfunction, and family support was significantly reduced. Patients who received chemotherapy received less social support from their extended family, and patients who received radiation therapy showed reduced seeking of spiritual support.

Patients felt that they received much less family support during all of their treatments than at the beginning of their illness diagnosis. Changes in post-operative measures indicated that the more patients increased family support, the more they sought social support, reframed and managed stress, and overall demonstrated better crisis management in their family. Considering the treatment type, family resilience and family support were negatively affected. In contrast, family dysfunction was not affected. The support that the patients received from both their family and their social environment resulted in better management of the disease.

## Figures and Tables

**Table 1:** Sociodemographic characteristics of the sample.

Variables		N	M/ Percentage %
Age		52.1 (10.3)	
Educational level			
	Primary school	2	3.4
	Middle school	5	8.6
	High school	14	24.1
	Post-secondary education	7	12.1
	Higher education	19	32.8
	Postgraduate education	7	12.1
	PhD	4	6.9
Employment Status			
	Unemployed	5	8.8
	Private employee	12	21.1
	Civil servant	17	29.8
	Housekeeping	9	15.8
	Entrepreneur	1	1.8
	Freelancer	3	5.3
	Other	10	17.5
Area of residence			
	<2000 inhabitants	4	6.9
	2000–10,000 inhabitants	7	12.1
	10,000–100,000 inhabitants	12	20.7
	>100,000 inhabitants	35	60.3
Nationality			
	Greek	57	98.3
	Other	1	1.7
Marital status			
	Unmarried	6	10.5
	Partnership	1	1.8
	Married	38	66.7
	Divorced	7	12.3
	Separated	2	3.5
	Widowed	3	5.3
Mothers			
	No	10	17.2
	Yes	48	82.8
Children’s gender			
	Male	15	3.6
	Female	17	37
	Male and Female	14	30.4
Number of participants who had underage children		20	34.4
Monthly income			
	Up to EUR 1000	30	55.6
	EUR 1001–1500	10	18.5
	EUR 1501–2000	10	18.5
	EUR 2001 and over	4	7.4
Family history of cancer			
	No	26	44.8
	Yes	32	55.2
Member of family with cancer diagnosis			
	Mother	6	19.4
	Father	7	22.6
	Siblings	5	16.1
	Grandparents	9	29
	Uncle/aunt	4	6.9
Person first informed of their cancer			
	Husband/Partner	33	56.9
	Father	0	0.0
	Mother	5	8.6
	Children	7	12.1
	Friends	6	10.3
	No one	2	3.4
	Other	3	5.2
	Many family members	2	3.4
Person closest to the patient			
	Husband/Partner	30	51.7
	Father	0	0.0
	Mother	4	6.9
	Children	14	24.1
	Friends	1	1.7
	No one	1	1.7
	Other	1	1.7
	Many family members	7	12.1

**Table 2:** Scores and changes in the studied scales, before and after the operation.

	N	Pre-Surgery	Post-Surgery	Change	Paired *t*-Test
		M (SD)	M (SD)	M (SD)	
Problem solving (FAD)	47	1.83 (0.39)	1.96 (0.46)	0.13 (0.44)	0.048
Communication (FAD)	46	1.92 (0.51)	1.97 (0.58)	0.05 (0.59)	0.599
Roles (FAD)	47	2.18 (0.45)	2.14 (0.41)	−0.04 (0.55)	0.632
Affective responses (FAD)	50	1.84 (0.52)	1.84 (0.7)	−0.1 (0.66)	0.944
Affective involvement (FAD)	48	1.95 (0.42)	2 (0.49)	0.05 (0.43)	0.450
Behavior control (FAD)	41	2.37 (0.39)	2.28 (0.37)	−0.09 (0.49)	0.262
General functioning (FAD)	45	1.76 (0.43)	1.79 (0.54)	0.03 (0.5)	0.694
Acquiring Social Support (F-COPES)	52	28.2 (6.4)	27 (7.7)	−1.1 (6.8)	0.240
Reframing (F-COPES)	49	33 (4.5)	32.7 (4.6)	−0.3 (3.9)	0.636
Seeking Spiritual Support (F-COPES)	57	12.6 (4.9)	13 (4.9)	0.4 (3.7)	0.482
Mobilizing Family to Acquire and Accept Help (F-COPES)	58	15 (3.8)	14.5 (3.7)	−0.5 (3.7)	0.321
Passive Appraisal (F-COPES)	56	12.3 (3.7)	12 (3.5)	−0.3 (3.1)	0.439
Overall F-COPES	44	102.8 (14.1)	101.6 (15.3)	−1.3 (13.1)	0.523
Family Problem Solving Communication (FPSC)	55	20.3 (4.1)	19.3 (4.4)	−1 (4.2)	0.097
Family support (FS-13)	47	63 (7.7)	51 (8)	−12 (6.9)	<0.001

**Table 3:** Changes in the F-COPES scale (sum scores), according to the type of treatment the patients received.

	Type of Treatment	Pre-Surgery	Post-Surgery	Change		
		M (SD)	M (SD)	M (SD)	P ^2^	P ^3^
Acquiring Social Support	Chemotherapy	28 (5.8)	25.1 (6.2)	−2.9 (5.6)	0.030	0.173
	Radiotherapy	29.3 (9.4)	27.4 (8.7)	−1.9 (3.2)	0.174	
	Hormone therapy	27.4 (4.6)	31 (6.4)	3.6 (10)	0.339	
	None	27.4 (4.9)	26.7 (8.9)	−0.6 (8.2)	0.802	
	P ^1^	0.915	0.317			
Reframing	Chemotherapy	32.1 (4.7)	31.5 (4.3)	−0.6 (3.6)	0.410	0.536
	Radiotherapy	35.5 (2.5)	34.3 (4.1)	−1.2 (3.3)	0.428	
	Hormone therapy	31.5 (4.6)	33.2 (6)	1.7 (7.6)	0.615	
	None	35.3 (3.2)	34.2 (4.2)	−1.1 (2)	0.104	
	P ^1^	0.038	0.162			
Seeking Spiritual Support	Chemotherapy	12.2 (5.2)	11.8 (4.9)	−0.5 (2.4)	0.369	0.122
	Radiotherapy	14.1 (6.7)	12.7 (6.4)	−1.4 (1.4)	0.035	
	Hormone therapy	13.3 (4.7)	16 (3.5)	2.8 (6.2)	0.249	
	None	12.4 (4.6)	13.2 (5)	0.8 (4.6)	0.558	
	P ^1^	0.828	0.229			
Mobilizing Family to Acquire and Accept Help	Chemotherapy	14.6 (3.5)	13 (4)	−1.6 (3.6)	0.043	0.188
	Radiotherapy	16.3 (2.5)	16 (1.9)	−0.3 (1.4)	0.626	
	Hormone therapy	16.3 (3.7)	16.8 (2.4)	0.5 (4.1)	0.743	
	None	14.5 (4.5)	15.4 (3.7)	0.8 (3.6)	0.416	
	P ^1^	0.507	0.029			
Passive Appraisal	Chemotherapy	12.3 (3.3)	12.7 (3.4)	0.3 (3.5)	0.637	0.338
	Radiotherapy	12.9 (4.9)	12.4 (3.5)	−0.5 (2.5)	0.590	
	Hormone therapy	12.5 (3)	10.5 (3.3)	−2 (2.9)	0.090	
	None	12.7 (3)	12 (3.4)	−0.7 (3)	0.452	
	P ^1^	0.979	0.457			
Overall F-COPES	Chemotherapy	100.1 (10.9)	95.3 (13.5)	−4.8 (11.3)	0.079	0.167
	Radiotherapy	112.4 (17.4)	107.6 (18.3)	−4.8 (7.9)	0.248	
	Hormone therapy	103.8 (16.2)	112.7 (10.5)	8.8 (21)	0.349	
	None	106.2 (13.4)	105.7 (15.6)	−0.5 (12.5)	0.902	
	P ^1^	0.236	0.045			

^1^ Difference between groups. ^2^ Difference between measures. ^3^ Repeated measures ANOVA. Differences in change between groups from one measure to another.

**Table 4:** Changes in the FS-13 and FPSC scales (sum scores), according to the type of treatment patients received.

	Type of Treatment	Pre-Surgery	Post-Surgery	Change		
		M (SD)	M (SD)	M (SD)	P ^2^	P ^3^
FS-13	Chemotherapy	65.3 (6.3)	52 (6.9)	−13.3 (6.6)	<0.001	0.458
	Radiotherapy	66.7 (7.9)	52.7 (8.4)	−14 (4.2)	<0.001	
	Hormone therapy	59.6 (9.2)	48.4 (8.2)	−11.1 (8.5)	0.013	
	None	61.7 (7.4)	52.2 (8.8)	−9.5 (8.1)	0.002	
	P ^1^	0.172	0.698			
FPSC	Chemotherapy	19.1 (4.4)	18.6 (4.2)	−0.5 (3.4)	0.479	0.976
	Radiotherapy	20.9 (3.2)	20.8 (3)	−0.1 (3.8)	0.928	
	Hormone therapy	21.3 (4.2)	20.9 (5)	−0.4 (3.8)	0.774	
	None	21.2 (2.8)	20.4 (2.8)	−0.8 (2.3)	0.260	
	P ^1^	0.298	0.354			

^1^ Difference between groups. ^2^ Difference between measures. ^3^ Repeated measures ANOVA. Differences in change between groups from one measure to another.

**Table 5:** Partial correlation coefficients of the changes in the FS-13 and FPSC scales with the change in the F-COPES scale, taking into account the type of treatment the participants received.

F-COPES: Pre-Post Comparison		FS-13	FPSC
Acquiring Social Support	R	0.55	−0.42
	P	0.064	0.177
Reframing	R	0.80	−0.14
	P	0.002	0.659
Seeking Spiritual Support	R	0.28	−0.44
	P	0.371	0.150
Mobilizing Family to Acquire and Accept Help	R	0.17	−0.29
	P	0.604	0.353
Passive Appraisal	R	−0.10	0.19
	P	0.755	0.555
Overall F-COPES	R	0.63	−0.45
	P	0.027	0.143

## Data Availability

The data are not publicly available due to confidentiality restrictions.
